# Supplementary material for: Quantifying dissipation using fluctuating currents
Source: Nat Commun. 2019 Apr 10;10:1666. doi: 10.1038/s41467-019-09631-x (PMC6458151; doi:10.1038/s41467-019-09631-x)
Supplement: Supplementary file 1 — Supplementary Information [file 41467_2019_9631_MOESM1_ESM.pdf]

Supplementary Information for "Quantifying dissipation using fluctuating currents"

Junang Li,[1] Jordan M. Horowitz,[1,2,3] Todd R. Gingrich,[1,4,*] and Nikta Fakhri[1,†]

[1] *Department of Physics, Massachusetts Institute of Technology, 77 Massachusetts Avenue, Cambridge, MA 02139*
[2] *Department of Biophysics, University of Michigan, Ann Arbor, Michigan, 48109*
[3] *Center for the Study of Complex Systems, University of Michigan, Ann Arbor, Michigan 48104*
[4] *Department of Chemistry, Northwestern University, Evanston, IL 60208*

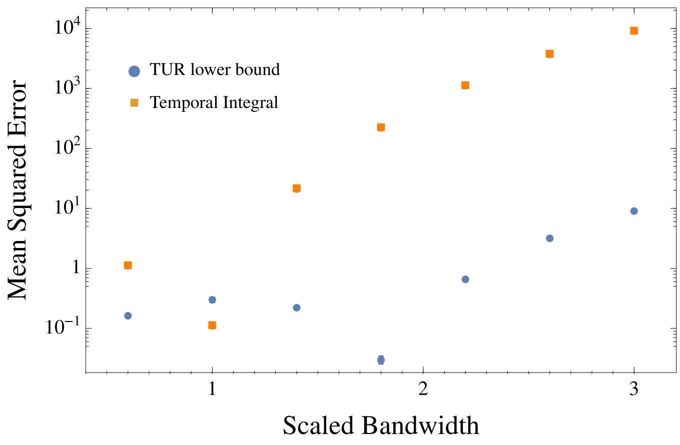

Supplementary Figure 1. **Selecting the bandwidth for kernel density estimation** The five-bead model's mean squared error for the temporal estimator (●) and the TUR lower bound (■) are sensitive to the bandwidth. Data are reported for $T_c/T_h = 0.1$ with the bandwidth scaled by the Bowman and Azzalini rule of thumb value. The MSE is estimated from Eq. (21) by averaging over ten independent trajectories with length $\tau_{obs} = 1200$. Error bars are the standard error, computed by repeating that procedure ten times.

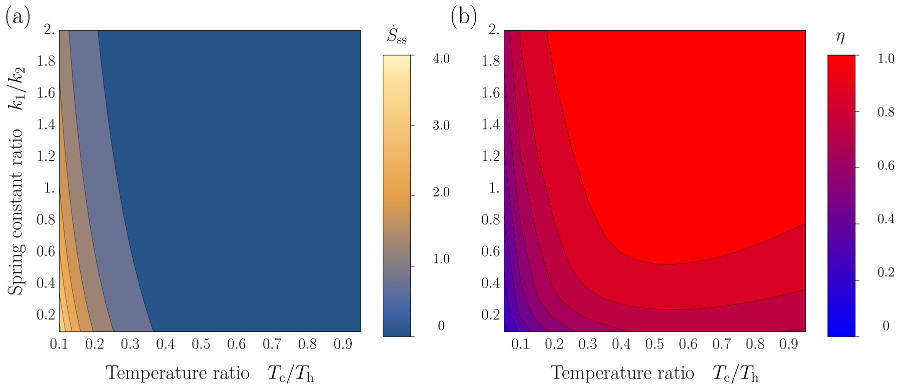

Supplementary Figure 2. **Efficiency of TUR bound estimation does not strictly improve with smaller dissipation** (a) The dissipation rate, measured in units of $k_B k_2/\gamma$, varies as a function of temperature ratio and spring constant ratio. In general $\dot{S}_{ss}$ increases with large temperature differences and spring constant differences. (b) The efficiency of the TUR lower bound calculated numerically using the tilting procedure. Notice that the contour lines of the two plots do not overlap, indicating that the microscopic details are also important to the tightness of the lower bound.

* todd.gingrich@northwestern.edu
† fakhri@mit.edu

SUPPLEMENTARY NOTES

Supplementary Note 1: Bead-spring analytical derivation

Solving for the steady-state behavior of linearly-coupled degrees of freedom is standard, but we review the derivation for completeness. As an ansatz, we insert a Gaussian steady state density, $\rho_{\rm ss} \propto e^{-\frac{1}{2}\mathbf{x}^T\mathcal{C}^{-1}\mathbf{x}}$, into the Fokker-Planck equation, where $\mathcal{C}$ is a symmetric matrix. It is straightforward to confirm from Eq. (3) that

$$\frac{\partial \rho_{\rm ss}(\mathbf{x})}{\partial t} = 0 \Rightarrow (A\mathcal{C} + \mathcal{C}A + 2D) = 0 \tag{1}$$

for symmetric A. We must then choose $\mathcal{C}$ such that the term in parentheses will vanish. The right choice is to set $\mathcal{C} = \lim_{t\to\infty} C(t)$, the long-time limit of the correlation matrix. To see the connection with the correlation matrix, note that the solution to a general Langevin equation of the form $\dot{\mathbf{x}} = A\mathbf{x} + F\boldsymbol{\xi}$ can be written as

$$\mathbf{x}(t) = \int_{-\infty}^{t} ds\ e^{A(t-s)} F \boldsymbol{\xi}(s) \tag{2}$$

for an arbitrary choice of A and F. Plugging Eq. (2) into the definition of correlation matrix $C_{ij}(t) = \langle x_i(0)x_j(t)\rangle$, one easily recovers Eq. (4). Differentiating Eq. (4) with respect to time gives $dC(t)/dt = AC(t) + C(t)A^T + 2D$, where we have used $D = FF^T/2$. The correlation matrix converges to a constant value at long times, so its derivative must vanish, requiring that $0 = A\mathcal{C} + \mathcal{C}A + 2D$. Hence the Gaussian ansatz solves the Fokker-Planck equation with $\mathcal{C}$ set by the correlation matrix. The steady-state current in Eq. (5) follows directly by plugging this $\rho_{\rm ss}$ into Eq. (3):

$$\mathbf{j}_{\rm ss}(\mathbf{x}) = A\mathbf{x}\rho_{\rm ss}(\mathbf{x}) - D\nabla\rho_{\rm ss}(\mathbf{x}). \tag{3}$$

The total entropy production rate is an integral of the local entropy production rate, which is the product of the current and the conjugate thermodynamic force

$$\dot{S}_{\rm ss} = \int d\mathbf{x}\ \mathbf{F}(\mathbf{x})\cdot\mathbf{j}_{\rm ss}(\mathbf{x}) = k_{\rm B}\int d\mathbf{x}\ \frac{\mathbf{j}_{\rm ss}^T(\mathbf{x})D^{-1}\mathbf{j}_{\rm ss}(\mathbf{x})}{\rho_{\rm ss}(\mathbf{x})}. \tag{4}$$

Inserting the formula for steady-state density and current from Eq. (5) into Supplementary Equation (4) yields

$$\dot{S}_{\rm ss} = k_{\rm B} \int d\mathbf{x}\ \mathbf{x}^T \left((A + \mathcal{C}^{-1}D)D^{-1}(A + D\mathcal{C}^{-1})\right)\mathbf{x}\ \rho_{\rm ss}(\mathbf{x}), \tag{5}$$

which simplifies to Eq. (7) upon performing the Gaussian integral.

In the main text, we discussed the steady-state properties with two beads, but the model with five beads could be solved following the same procedure. In this case, A and D are 5×5 matrices:

$$A = \begin{pmatrix} -2k/\gamma & k/\gamma & 0 & 0 & 0 \\ k/\gamma & -2k/\gamma & k/\gamma & 0 & 0 \\ 0 & k/\gamma & -2k/\gamma & k/\gamma & 0 \\ 0 & 0 & k/\gamma & -2k/\gamma & k/\gamma \\ 0 & 0 & 0 & k/\gamma & -2k/\gamma \end{pmatrix} \tag{6}$$

$$D = \frac{k_{\rm B}}{4\gamma}\begin{pmatrix} 4T_{\rm h} & 0 & 0 & 0 & 0 \\ 0 & 3T_{\rm h}+T_{\rm c} & 0 & 0 & 0 \\ 0 & 0 & 2T_{\rm h}+2T_{\rm c} & 0 & 0 \\ 0 & 0 & 0 & T_{\rm h}+3T_{\rm c} & 0 \\ 0 & 0 & 0 & 0 & 4T_{\rm c} \end{pmatrix}. \tag{7}$$

The total entropy production rate, calculated from the first line of Eq. (7), simplifies to

$$\dot{S}_{\rm ss} = k_{\rm B}\frac{k(T_{\rm h}-T_{\rm c})^2(111T_{\rm h}^2 + 430T_{\rm h}T_{\rm c} + 111T_{\rm c}^2)}{495T_{\rm h}T_{\rm c}(3T_{\rm h}+T_{\rm c})(T_{\rm h}+3T_{\rm c})\gamma} \tag{8}$$

for the five-dimensional case.

Supplementary Note 2: Lower Bound Efficiency

As discussed in the main text, the tightness of the TUR lower bound $\eta \equiv \dot{S}^{(\mathbf{F})}_{\mathrm{TUR}}/\dot{S}_{\mathrm{ss}}$ cannot be simply understood as a function only of the thermodynamic driving force. To illustrate that this inference efficiency η depends on microscopic details of the model, we examine the situation that the inter-bead and bead-wall coupling constants are not equal. In other words, the spring constants showing in Fig. 1 are replaced, from left to right, by k_1, k_2, and k_1. Consequently, the elastic coupling tensor A becomes

$$A = \begin{pmatrix} -(k_1+k_2)/\gamma & k_2/\gamma \\ k_2/\gamma & -(k_1+k_2)/\gamma \end{pmatrix}, \tag{9}$$

while the diffusion tensor D is the same as in the main text. Using Supplementary Equation (1) and Eq. (7), it is straightforward to obtain the total entropy production rate

$$\dot{S}_{\mathrm{ss}} = k_{\mathrm{B}} \frac{k_2^2 (T_{\mathrm{h}} - T_{\mathrm{c}})^2}{2(k_1+k_2)\gamma T_{\mathrm{h}} T_{\mathrm{c}}}. \tag{10}$$

In Supplementary Figure 2(a), we plot this entropy production rate as a function of the dimensionless ratios k_1/k_2 and $T_{\mathrm{c}}/T_{\mathrm{h}}$, illustrating the monotonic increase in entropy production as the spring constants or temperatures become more unequal.

We sought to understand how the TUR bound efficiency η depends on these same dimensionless constants. Using the tilting procedure, we numerically extracted $\dot{S}^{(\mathbf{F})}_{\mathrm{TUR}}$ and computed η, plotted in Supplementary Figure 2(b). We anticipated that the TUR bound would be tightest near equilibrium ($T_{\mathrm{c}}/T_{\mathrm{h}} \to 1$), but we observed that the spring constant ratio also strongly influences η. Interestingly, the contour plots for η bears a strong resemblance to that of $\dot{S}_{\mathrm{ss}}$ for small temperature ratios. In that regime, the TUR bound tends to become weaker as the dissipation rate increases. But that trend is not true in general. Contour lines of Supplementary Figure 2(a) trace spring constant and temperature ratios that hold the dissipation rate constant. While these contours roughly line up with the contours of η at small $T_{\mathrm{c}}/T_{\mathrm{h}}$, they deviate close to equilibrium, highlighting that the entropy production rate does not generically provide insight into the tightness of the TUR bound. Microscopic details, in this case the ratio of spring constants, are also important.
